# Editorial: Physiological and molecular mechanisms of important agronomic traits in plants under various abiotic factors

**DOI:** 10.3389/fpls.2024.1502061

**Published:** 2024-10-18

**Authors:** Zhibo Wang, Dominik K. Großkinsky, Dongmei Li, Weiwei Zheng

**Affiliations:** ^1^ School of Plant and Environmental Sciences, Virginia Tech, Blacksburg, VA, United States; ^2^ Bioresources Unit, Center for Health and Bioresources, AIT Austrian Institute of Technology, Tulln, Austria; ^3^ College of Horticulture Science and Engineering, Shandong Agricultural University, Taian, China; ^4^ State Key Laboratory of Crop Biology, Shandong Agricultural University, Taian, China; ^5^ Key Laboratory of Quality and Safety Control for Subtropical Fruit and Vegetable, Ministry of Agriculture and Rural Affairs, Collaborative Innovation Center for Efficient and Green Production of Agriculture in Mountainous Areas of Zhejiang Province, College of Horticulture Science, Zhejiang A&F University, Hangzhou, China

**Keywords:** abiotic stress, physiological response, molecular mechanism, agronomic trait, global climate change

The effects of abiotic stress on plant growth, yield, and quality have become increasingly significant in light of global climate change. In this Research Topic, we explore the physiological and molecular mechanisms by which plants respond to various environmental stressors, such as drought, temperature extremes, salinity, metal, chemical, and pollutant exposure. It compiles ten original research articles that provide new insights into how plants adapt and maintain critical agronomic traits in the face of adverse environmental conditions.

## Understanding plant responses to abiotic stresses

Plants face an array of environmental challenges, including extreme temperatures, water scarcity, high salinity, and more, all of which negatively impact productivity. These abiotic stresses disrupt cellular homeostasis and alter metabolic pathways, leading to reduced growth, development, and yield. In response, plants have evolved various physiological mechanisms to mitigate these effects. Understanding and evaluating these physiological changes provide critical phenotypic data for investigating genetic mechanisms, which can ultimately aid in molecular breeding efforts.

Two original research studies in this Research Topic focus on plant responses to abiotic stress. Wang et al. explore the response of alfalfa (*Medicago sativa* L.) root systems using a metabolomics approach. They identify amino acids, organic acids, sugars, and alkaloids as key metabolites essential for alfalfa’s resistance to drought, including compounds such as 6-gingerol, salicylic acid (SA), indole-3-acetic acid (IAA), gibberellin A4 (GA4), abscisic acid (ABA), trans-cinnamic acid, sucrose, L-phenylalanine, L-tyrosine, succinic acid, and nicotinic acid.

Davoudi et al. investigate the effects of elevated CO_2_ levels on the physiology of strawberries and tomatoes. With atmospheric CO_2_ levels having increased by more than 20% in the past four decades, this research is highly significant. They found that a three-month exposure to 800 ppm CO_2_ increased yields in both strawberry and tomato. Additionally, while both species prioritized fruit development over other sink organs, they were limited by carbon export at elevated CO_2_ levels, as new photoassimilates were evenly distributed across various sinks, regardless of CO_2_ conditions.

## Advances in molecular biology, genetics, and genomics related to abiotic stress adaptation

With food security becoming an increasingly pressing concern, breeding stress-resistant crops has emerged as a critical focus in modern agriculture. This effort is bolstered by advancements in our understanding of the molecular mechanisms and genetic components involved in signaling cascades, transcriptional networks, structural modifications, and biochemical pathways. This Research Topic presents groundbreaking studies that advance molecular biology, genetics, and genomics associated with abiotic stress tolerance, enhancing our ability to dissect plant stress responses.

Tian et al. cloned the *CabHLH18* gene, a specific bHLH transcription factor, from a waterlogging-tolerant pepper cultivar, ‘ZHC2’. Compared with wild-type (WT) plants, pepper plants overexpressing *CabHLH18* showed greater water content, amino acid, proline, soluble sugar levels, root viability, and superoxide dismutase activity, while exhibiting lower malondialdehyde content under waterlogging conditions. After 24 hours of waterlogging stress, the fresh weight, amino acid, proline, and soluble sugar levels of the overexpression lines were higher than those of the WT plants. Hence, *CabHLH18* is a promising candidate for breeding waterlogging-tolerant hot pepper varieties.

Liu et al. investigated respiratory burst oxidase homologs (RBOHs), a key enzyme family regulating superoxide production and playing a central role in plant stress responses. Seven *PsRBOH* genes were identified in the pea genome, with tissue-specific expression patterns and functional diversity during growth and stress responses. *PsRBOH4* emerged as a key responsive gene, as its expression was significantly induced under heat, cold, cadmium, drought, and low boron stresses, while *PsRBOH1* primarily responded to salt stress. This study provides valuable insights into the functional roles of pea *RBOH* genes in plant adaptation to climate-related challenges.

Aluminum (Al) toxicity in acidic soils is a major limiting factor affecting crop yield, inhibiting root growth, reducing nutrient and water absorption, and ultimately impairing photosynthesis. Zhang et al. studied potato aluminum-activated malate transporters (ALMTs), which play important roles in responding to Al toxicity, maintaining ion homeostasis, and supporting mineral nutrient distribution. Fourteen *StALMT* genes were identified in the potato genome, unevenly distributed across seven chromosomes. Specific *StALMT* genes were significantly up-regulated in response to Al^3+^ and overexpression of these genes conferred enhanced growth resistance to Al toxicity, highlighting the pivotal role of these genes in combating Al^3+^ toxicity in plants.

Khassanova et al. identified two chickpea zinc finger knuckle genes, *Ca04468* and *Ca07571*, as key candidates in plant responses to drought and dehydration. Various methods, including Sanger sequencing, DArT (Diversity Array Technology) for plant genotyping, molecular marker and gene expression analyses, and field trials, were used to characterize these genes. Associations with 100-seed weight and seed weight per plant were examined, and two SNP molecular markers for both genes were developed and verified. These markers hold potential for marker-assisted selection to improve drought and dehydration tolerance in chickpea, paving the way for the development of novel chickpea cultivars in the future.

These studies collectively highlight the critical advancements in understanding the molecular, genetic, and genomic bases of abiotic stress resistance, providing invaluable tools and knowledge for breeding climate-resilient crop varieties.

## Cross-talk between stress responses pinpoints key genetic regulators that serve as central hubs for conferring multiple stress resistance

Under changing climatic conditions, crop plants are increasingly affected by combinations of multiple abiotic stresses rather than individual stress factors. Studies in this Research Topic explore the concept of cross-talk between different abiotic stress responses. Samarina et al. investigate the role of specific transcription factors in regulating stress-responsive genes in tea plants under drought and cold conditions, as well as gene models related to cell wall remodeling. The highlighted key signaling pathways suggest that plants employ shared mechanisms to cope with multiple stressors, providing a molecular basis for breeding tea plants with enhanced tolerance to interactive abiotic stresses.

The frequent co-occurrence of various abiotic stresses highlights the need to identify potential donors resistant to multiple stressors for developing climate-resilient crop varieties. In this context, Kumar et al. screened 41 rice germplasm accessions, including landraces and elite cultivars, for tolerance to drought, salinity, and submergence at the 21-day-old seedling stage over a 10-day period. Specific genotypes were identified as promising donors for multiple abiotic stress tolerance. Additionally, a set of 30 SSR markers linked to drought, salinity, and submergence QTLs were used to characterize these accessions, providing valuable genomic tools for future breeding efforts.

## New insights into interactions between plants and atypical abiotic stress

Continuous agricultural production can also introduce atypical abiotic stress to plants. Zhou et al. reported that continuous cropping of tobacco results in the accumulation of allelopathic compounds in the rhizosphere. Redundancy analysis (RDA) identified eight compounds with autotoxic effects on tobacco growth. These compounds contributed to yield reductions, outbreaks of tobacco black shank, and a decline in beneficial soil flora.

Expanding industrialization and other human activities lead to an increase in the occurrence of sudden environmental pollution accidents (SEPAs), an atypical form of abiotic stress. Developing methods to promptly eliminate pollutants at their source and address the resulting environmental issues is crucial for global ecological health and sustainable human development. Phytoremediation, a biological approach, offers advantages such as simplicity, cost-effectiveness, and the reduction of secondary pollution compared to traditional methods. Li et al. developed a 3D-QSAR pharmacophore model to predict plant resistance and the phytodegradation of polychlorinated biphenyls (PCBs), a class of organic pollutants regulated under the Stockholm Convention due to their persistence, high toxicity, bioaccumulation, and long-range environmental transport. This study provides theoretical support for the application of transgenic plant-based emergency phytoremediation technology.

## Future perspectives

As the global climate continues to change, the frequency and intensity of abiotic stresses will likely increase, further challenging global food production systems. This Research Topic provides a foundation for developing stress-tolerant crops that can thrive in adverse conditions. Future work should focus on validating the newly identified molecular mechanisms underlying the physiological responses, integrating these insights into breeding programs and agricultural practices, ensuring that crop yield and quality can be maintained in the face of environmental uncertainties. This Research Topic represents a significant step toward understanding the intricate mechanisms that govern plant stress responses and offers promising avenues for improving crop resilience through innovative biotechnological approaches.

